# Diffusion Tractography in Deep Brain Stimulation Surgery: A Review

**DOI:** 10.3389/fnana.2016.00045

**Published:** 2016-05-02

**Authors:** Evan Calabrese

**Affiliations:** Center for In Vivo Microscopy, Department of Radiology, Duke University Medical CenterDurham, NC, USA

**Keywords:** diffusion tractography, deep brain stimulation, neuroanatomy, magnetic resonance imaging, tractography, diffusion tensor imaging

## Abstract

Deep brain stimulation (DBS) is believed to exert its therapeutic effects through modulation of brain circuitry, yet conventional preoperative planning does not allow direct targeting or visualization of white matter pathways. Diffusion MRI tractography (DT) is virtually the only non-invasive method of visualizing structural connectivity in the brain, leading many to suggest its use to guide DBS targeting. DT-guided DBS not only has the potential to allow direct white matter targeting for established applications [e.g., Parkinson’s disease (PD), essential tremor (ET), dystonia], but may also aid in the discovery of new therapeutic targets for a variety of other neurologic and psychiatric diseases. Despite these exciting opportunities, DT lacks standardization and rigorous anatomic validation, raising significant concern for the use of such data in stereotactic brain surgery. This review covers the technical details, proposed methods, and initial clinical data for the use of DT in DBS surgery. Rather than focusing on specific disease applications, this review focuses on methods that can be applied to virtually any DBS target.

## Introduction and Background

Deep brain stimulation (DBS) has become an established therapy for medically refractory movement disorders including Parkinson’s disease (PD), essential tremor (ET), and dystonia. DBS is also currently under investigation for use in a variety of other neurologic and psychiatric conditions including depression, chronic pain, and obsessive compulsive disorder.

Diffusion MRI tractography (DT) refers to 3D models of white matter pathways generated from diffusion weighted MRI data, most commonly diffusion tensor imaging (DTI). Here, the term DT is used to refer to all forms of tractography derived from diffusion MRI data including but not limited to DTI (Mori et al., 1999), Q-ball (Tuch, 2004; Descoteaux et al., 2007), constrained spherical deconvolution (CSD; Tournier et al., 2008), BEDPOSTX (Behrens et al., 2007), and diffusion spectrum imaging (DSI; Wedeen et al., 2005). DT is currently the only non-invasive method for modeling structural brain connectivity in humans.

This review covers recent work on integrating DT into DBS surgical planning. Several facets of this complex topic have been previously reviewed (Henderson, 2012; Torres et al., 2014). The current review differs from previous work in that it focuses on DT methods in DBS surgery, and associated technical concerns, rather than on their use for the treatment specific diseases. In addition, it incorporates new research that was published in the approximately 2 years since the most recent review was written. Nonetheless, previous reviews are valuable counterparts to this work, particularly for researchers with interest in a specific disease.

As the use of DBS increases both in scope and in patient numbers, there is a need to evaluate and improve each step of the surgical process. Several groups have suggested and even implemented a variety of DT-based DBS targeting techniques (Coenen et al., 2011a; Hunsche et al., 2013; Schlaepfer et al., 2013). The rationale for incorporating DT into DBS planning is based on two prominent, though not rigorously proven theories in DBS research. First, that DBS functions, at least in part, by modulation of neural circuitry, and second, that direct targeting of the circuitry on which DBS is believed to exert its effects will improve patient outcomes (Coenen et al., 2012).

**Figure 1:**
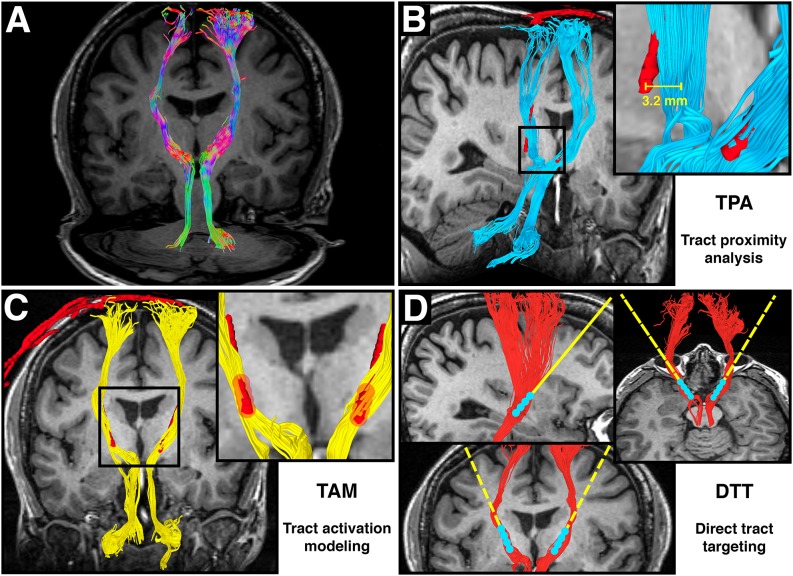
**Adapted from Calabrese et al. (2015b) Figure 6, with permission. (A)** Directionally colored diffusion MRI tractography (DT) of the dentatorubrothalamic tract (DRT), which connects the dentate nucleus of the cerebellum, red nucleus, ventral intermediate thalamus, and motor cortex. Note the absence of a midline crossing in the midbrain, which is a limitation of the diffusion tensor imaging (DTI) technique. **(B)** Tract proximity analysis (TPA). Distances between the DRT (blue) and the deep brain stimulation (DBS) electrode (red) are compared with treatment outcomes. **(C)** Tract activation modeling. Fiber tracts (yellow) are generated from a region of interest surrounding the DBS contacts (red). **(D)** Direct tract targeting (DTT). The DT model of the DRT (red) is used for preoperative DBS electrode targeting.

Unlike its predecessor, ablative brain lesioning, DBS theoretically leaves brain connections intact. Due to the similar efficacy of DBS and brain lesioning for movement disorders, it was initially thought that DBS functioned by creating a “functional lesion” via continuous depolarization lock of local neurons (Benabid et al., 2002). While appealing as a heuristic, the functional lesion theory fails to explain several aspects of DBS including, for example, the fact that lesions of the globus pallidus externus produce Parkinsonism, while DBS of the same region can reverse Parkinsonian symptoms (Vitek et al., 2004). High-frequency stimulation does appear to inhibit local neurons, but there is also evidence that it simultaneously produces downstream excitatory activity (Hashimoto et al., 2003; McIntyre et al., 2004a). In fact, evidence from a variety of different sources including functional MRI, optogenetics, and DT connectivity analysis suggests that DBS may exert effects in sites distant from, but structurally connected to, stimulation sites (McIntyre et al., 2004b; Gradinaru et al., 2009; Schweder et al., 2010). Exactly how DBS exerts therapeutic effects through neurocircuitry is less clear but several theories exist. For example, substantial evidence suggests that ET is caused by aberrant neural activity in the dentatorubrothalamic tract (DRT; Coenen et al., 2014), a pathway connecting the dentate nucleus of the cerebellum to the contralateral red nucleus, ventral intermediate thalamus, and motor cortex (Figure 1A). It has therefore been suggested that DBS for ET functions through normalization of, or indirect inhibition of, pathological activity (Chiken and Nambu, 2015). It is also possible that DBS causes global brain changes that extend beyond the area of stimulation and immediate downstream connections (Montgomery and Gale, 2008). For example, experiments on DBS of the subgenual cingulum for depression have demonstrated both modulation of neural activity and changes in gene expression at various distant cortical sites, many of which are not directly connected to the stimulation region (Lujan et al., 2013; Riva-Posse et al., 2014). The exact mechanisms of DBS remain largely unknown and are more thoroughly discussed elsewhere (Benabid et al., 2002; Montgomery and Gale, 2008). However, it is clear that white matter connections play a role in DBS, and exploring this role will be an important area for future research.

Interestingly, while surgical treatment for movement disorders has evolved from ablation to DBS-mediated neuromodulation, stereotactic targeting methods have not followed suit. Conventional DBS preoperative planning uses essentially the same stereotactic targeting methods as ablative procedures. While conventional stereotactic targeting methods have served functional neurosurgeons for decades, they have two major drawbacks that limit their use for DBS targeting. First, stereotactic coordinates are often derived from histology-based human brain atlases, which are 2D and prone to spatial distortions from fixing, sectioning, and staining of tissue slices. Second, available targets consist largely of brain nuclei, and direct targeting or visualization of white matter pathways is typically not possible. If indeed DBS exerts its therapeutic effects through direct modulation of neural circuitry, it stands to reason that DBS targeting should be focused on neural circuits. There are many potential benefits of direct targeting of neurocircuitry for DBS. First, it may improve targeting accuracy, allowing fewer passes, fewer ineffective surgeries, and perhaps even obviate the need for intraoperative contact stimulation testing. Second, it may expand DBS therapy to new diseases by allowing targeting of structures that are not visible using conventional methods. Finally, it may aid in our understanding of the mechanisms of DBS by revealing the exact pathways affected by stimulation.

To date, DT remains the only non-invasive method for visualizing human brain connections. DT suffers from both fundamental and practical limitations that limit its use for modeling brain connections. Unlike many invasive modalities, DT is incapable of determining the direction of information flow, nor can it distinguish single- and multi-neuron connections. DT may also have difficulty resolving complex intra-voxel fiber crossings or non-dominant fiber populations due to limitations in scan time, hardware, or processing methods. Despite its many limitations, DT has been successfully used to model human neuronal connections for over two decades, including several pathways that are putative DBS targets (Sedrak et al., 2008).

This review contains two main discussion sections. The first addresses technical considerations for designing DT-based studies in DBS patients. This section includes discussion of the details of diffusion data acquisition, preprocessing, and DT generation. The second is a review of recent studies on integrating DT into DBS surgery. This is not meant to be an exhaustive analysis of DT-based DBS studies, but rather a broad overview of common methods and their applications. A majority of these studies can be divided into three broad groups based on the primary methodology. In this review, we will refer to these broad methodologies as: Tract stimulation modeling (TSM); Tract proximity analysis (TPA); and Direct tract targeting (DTT). Figures 1B–D show graphic representations of these three research techniques. Each method has a different goal, and each will be discussed separately. Throughout this review, implantable quadripolar brain stimulator leads will be referred to as “electrodes”, while the individual stimulation elements will be referred to as “contacts”. DT based models of white matter pathways will be referred to as “tracts”. Table 1 provides a list of abbreviations used throughout the manuscript.

**Table 1:** **List of abbreviations used in this review**.

Abbreviation	Definition
CSD	Constrained spherical deconvolution
DBS	Deep brain stimulation
DRT	Dentatorubrothalamic tract
DSI	Diffusion spectrum imaging
DT	Diffusion tractography
DTI	Diffusion tensor imaging
DTT	Direct tract targeting
ET	Essential tremor
HARDI	High angular resolution diffusion imaging
MRI	Magnetic resonance imaging
PD	Parkinson’s disease
SNR	Signal-to-noise ratio
TPA	Tract proximity analysis
TSM	Tract stimulation modeling

## Technical Considerations

DT generation can be divided into three separate steps: data acquisition, data processing, and tracking. Each of these steps has several variables that must be considered in order to ensure accurate DT. Many of the most common and important variables are discussed here.

### Data Acquisition

Data acquisition involves the collection of diffusion weighted MRI data from subjects. For DT studies, the most important acquisition parameters include image resolution, diffusion weighting factor (*b*-value), and the number and distribution of diffusion measurements. Image resolution is important for DT because some fiber configurations, such as intra-voxel curving, can only be resolved by increasing spatial resolution (Calabrese et al., 2014). Image resolution also affects the accuracy of tract volume estimates, which is essential for DT-based DBS targeting (Lebel et al., 2011). In clinical MRI, resolution is typically limited by signal-to-noise ratio (SNR), and it is uncommon to achieve voxel sizes smaller than 2 mm isotropic at 3T. Many studies report spatial resolutions of 1 mm or less in plane, but typically have much larger (e.g., 3 mm) slice thickness. The use of anisotropic voxels should be discouraged for DT studies as it complicates accurate determination of fiber angles and fiber crossings due to partial volume effects (Mukherjee et al., 2008). SNR, and therefore resolution, can be improved by averaging or repeating scans, but a doubling of total scan time only increases SNR by a factor of √2 or approximately 1.4. Other important yet infrequently mentioned parameters that contribute to effective image resolution are sense factor, and incomplete *k*-space acquisition strategies like partial Fourier acquisition or zerofilling in *k*-space (Paschal and Morris, 2004). In general, it is best to use the highest isotropic resolution achievable without compromising SNR, and within a scan time compatible with clinical constraints (Jones and Cercignani, 2010). Increased resolution is unlikely to reduce accuracy, but it may not always be beneficial. For example, McNab et al. (2009) showed good correspondence between standard 2 mm isotropic preoperative DT and 0.73 mm isotropic postmortem DT in a patient who received DBS of the subgenual cingulum. In DBS studies, image resolutions as high as 1.6 mm isotropic have been used (Sudhyadhom et al., 2012).

In addition to spatial resolution, diffusion MRI requires consideration of intravoxel *diffusion* resolution. All DT techniques, in essence, use measurements of the 3D diffusion function in each voxel (often referred to as the diffusion propagator) to determine underlying fiber orientation. By convention, MRI measures diffusion in a spherical coordinate system known as *q*-space, which has a Fourier transform relationship with the diffusion propagator. All points in *q*-space can be defined by two angles (the diffusion measurement direction) and a radius (the diffusion weighting factor, or *b*-value). Increasing the number of diffusion measurement directions increases the angular resolution of the fiber reconstruction, which can allow multiple fibers to be reconstructed from a single voxel with appropriate processing. In general, higher *b*-values yield a larger signal difference between restricted and unrestricted diffusion at the cost of lower SNR. This increased signal difference affects the ability to accurately detect certain fiber populations (Frank, 2001; Basser, 2002). Both the number and the arrangement of *q*-space measurements required for accurate fiber reconstruction depends heavily on the constraints and assumptions of the DT technique being used.

The simplest case, DTI, assumes that there is a single fiber population, and that the diffusion propagator is a tensor. As such, it requires only six unique diffusion measurement directions and a *b*-value sufficient to distinguish the primary diffusion direction from the two perpendicular diffusion directions (usually *b* ≥ 800 s/mm^2^). In practice, using only six diffusion measurements is problematic because the reconstruction algorithm is highly sensitive to error with minimal inputs (Lebel et al., 2011). Increasing the number of diffusion measurement directions improves the accuracy of DTI fiber orientation estimations, but diminishing returns are reached at around 30 unique directions (Jones, 2004). Similarly, for DTI low *b*-values can introduce error, but there is little benefit to increasing *b*-value above *b* = 1500 s/mm^2^ (Dyrby et al., 2011). In contrast, multi-fiber methods such as spherical harmonic Q-ball (Descoteaux et al., 2007), have far fewer model assumptions, and therefore require more diffusion measurement angles and higher *b*-values. Typical values are 30–120 directions and *b-values* in the 2000–5000 s/mm^2^ range. Diminishing returns are reached at around 90 directions and *b* = 4000 s/mm^2^ (Tournier et al., 2013). DSI, a DT reconstruction method that is often described as “model free”, requires hundreds of diffusion measurement angles and *b*-values in the 10,000–50,000 s/mm^2^ range because it attempts to directly calculate the underlying diffusion propagator in each voxel using the Fourier transform relationship (Wedeen et al., 2008). *b-values* in this range are often difficult or impossible to achieve on certain scanners because of hardware and/or SNR limitations. Even if appropriate hardware is available, lengthy diffusion sampling schemes may be impractical or cost-prohibitive in DBS patients. In general, diffusion sampling scheme should be chosen based on the data processing method, and should be as complete as possible given scan time limitations. In the existing DBS literature, 12–60 diffusion directions have been used, typically at *b* = 1000 s/mm^2^ (Johansen-Berg et al., 2008; Klein et al., 2012; Sudhyadhom et al., 2012; Anthofer et al., 2014).

### Data Processing

There are many different DT data processing methods available, each with different requirements, assumptions, limitations and benefits. Perhaps the most important processing difference for DBS studies is single-fiber (e.g., DTI) vs. multi-fiber reconstruction (e.g., Q-ball; Tuch, 2004). Multi-fiber methods may be preferable since many putative tract targets for DBS are located in structurally complex brain regions. Further, it has been shown that at clinical image resolution, 60–90% of white matter voxels contain crossing fibers (Jeurissen et al., 2012). Another important processing difference is direct fiber estimation methods (e.g., BEDPOSTX, CSD) vs. orientation distribution function-based methods (e.g., Q-ball, DSI). Orientation distribution function based methods attempt to reconstruct the diffusion propagator in each voxel, and then infer fiber orientation from the peaks of this function. In contrast, direct fiber estimation methods attempt to recover fiber orientation directly from diffusion MRI data. Direct methods may be less prone to error, and more flexible in terms of sampling requirements, but often have dramatically increased computational requirements (Behrens et al., 2007; Tournier et al., 2008, 2013).

DTI is by far the most common DT method used in DBS studies (Torres et al., 2014). This technique has the advantage of being readily available on most commercial scanners, as well as having a relatively quick acquisition, and a simple reconstruction. The major drawbacks of this method are the assumptions of the model and the fact that it only accounts for a single fiber population per voxel, which is insufficient for a majority of voxels in the human brain (Jeurissen et al., 2012). The strengths and weaknesses of other fiber reconstruction methods like Q-ball, CSD, and DSI are less clear, and depend heavily on the diffusion measurement scheme and the particular fiber tract of interest (Calabrese et al., 2014). These advanced DT methods are almost certainly more anatomically accurate than DTI, but their sampling requirements may be prohibitive in DBS patients, and it is not known if the added anatomic accuracy actually improves DBS surgical planning or electrode targeting. It is also important to note that many tractography data processing methods are neither designed for nor approved for use in surgical planning. Nonetheless, as DBS targets become more complex and nuanced, it stands to reason that advanced DT processing methods may play an important role in accurately visualizing the underlying anatomy. For instance, there are several examples of DBS studies that utilize BEDPOSTX fiber reconstruction for complex anatomic targets (Johansen-Berg et al., 2008; Klein et al., 2012; Clelland et al., 2014; Choi et al., 2015).

### Tracking

Even with the exact same data and processing method, differences in tracking algorithms can dramatically affect the size, shape, and extent of resultant tracts, which has obvious implications for DT-based DBS targeting (Fillard et al., 2011). Most of the differences in tracking algorithms can be distilled to deterministic vs. probabilistic tracking, and the choice of tracking thresholds. Deterministic DT algorithms use only the calculated fiber orientations, while probabilistic algorithms randomly draw from a probability distribution of fiber orientations based on estimated error. Deterministic algorithms thus generate the same set of tracts every time, while probabilistic methods yield a random set of probability distributed tracts. This random, iterative process makes probabilistic methods much more computationally expensive and time consuming. The data formats for probabilistic and deterministic DT are also quite different, and often require different analysis methods. Deterministic data are typically stored as a set of 3D streamlines, while probabilistic data usually takes the form of an image, where intensity values reflect strength or probability of connection between a given voxel and a seed region. Probabilistic DT data can be easily adjusted using thresholding, which adds an additional layer of complexity. For example, contact between a DBS electrode and deterministic fiber tract is all or none, while contact with a probabilistic tract can be expressed in terms of contact strength or probability (Pouratian et al., 2011). In addition, probabilistic methods can be more sensitive than deterministic methods for non-dominant fiber pathways, but are also likely to have more false positives (Behrens et al., 2007). The choice of deterministic vs. probabilistic DT depends on the application, but for complex pathways probabilistic methods may be preferable (Behrens et al., 2007; Kwon et al., 2011). Although deterministic tractography is significantly more common for DBS applications, there are a number of published studies that use probabilistic methods (Johansen-Berg et al., 2008; Pouratian et al., 2011; Sudhyadhom et al., 2012; Riva-Posse et al., 2014).

The choice of DTI tracking thresholds (e.g., angle threshold, fractional anisotropy threshold) also depends on the application, and should generally be tailored to the specific tract of interest. For example, DT of cortical areas, or regions with many crossing fibers requires a very low fractional anisotropy threshold, and tracts with high curvature obviously require larger angle thresholds.

There are several other important differences between different tracking algorithms, particularly with regard to how tracts are propagated. The most basic tracking algorithms follow the calculated fiber orientation exactly when moving from voxel to voxel, while others integrate fiber direction over several adjacent voxels (Mori et al., 1999; Lazar et al., 2003). These differences affect anatomic accuracy and propagation of error, and can yield wildly different tracts even when performed on the same dataset (Lazar and Alexander, 2003; Fillard et al., 2011).

The choice of what to track is also important, particularly for DBS applications. If a pathway is known *a priori*, it may be possible to develop a standard set of seed and waypoint regions to allow reliable tracking (Coenen et al., 2011c, 2012; Anthofer et al., 2015). If the goal is to investigate the pathways being affected by given DBS electrode, a seed region can be generated around the implantation site using co-registered pre- and post-operative imaging. Such seed regions can range from simple cubes (Barkhoudarian et al., 2010) to elaborate electric field models based on stimulation parameters and the electrical properties of surrounding tissue (Butson et al., 2006, 2007). While electric field modeling adds considerable complexity to a study, it might also provide a more accurate estimation of the fiber populations that are modulated by DBS electrodes.

Another major issue with tracking is the lack of full-featured software suite for tracking and surgical planning in the surgical context. Many of the techniques described here, including probabilistic tractography and electric field modeling, are not commonly available in surgical planning software packages. In the absence of clinically tested or approved software, these techniques remain primarily research tools.

### Anatomic Accuracy

Recently, several groups have shown that DT has relatively poor anatomic accuracy, particularly in areas of neuronal complexity (Thomas et al., 2014; Calabrese et al., 2015a). DT has both practical and fundamental limitations that decrease its anatomic accuracy. From a practical standpoint, diffusion MRI data derived from human subjects is never ideal due to factors like scan time considerations, hardware limitations, patient motion, cardiac pulsation, and bulk flow. Each of these factors has the potential to affect the anatomic accuracy of DT by introducing error into DT processing algorithms. Even in the absence of practical concerns, there are fundamental limitations to the DT method (Thomas et al., 2014). First, DT is based on the assumption that the diffusion of water in the brain follows axonal pathways, which may not always be correct. Second, DT is constrained by the assumptions of the reconstruction and tracking models. For example, many reconstruction algorithms assume that water diffusion in the brain is Gaussian in nature, and there is often an implicit or explicit limit in the number of fiber populations that can be reconstructed from a single voxel. Even so-called “model free” fiber reconstruction methods are still subject to the assumptions of tracking algorithms including tract initiation and termination criteria, and step length (Wedeen et al., 2008). Fortunately, for DBS targeting applications, the absolute accuracy of DT is not important as long as it is accurate enough to guide effective electrode placement. This question, “is DT accurate enough for effective DBS targeting?” is one of the fundamental motivations for the studies reviewed in the next section.

## DT-Based DBS Methods

A number of different studies have been conducted with the goal of incorporating DT into DBS surgery. Table 2 is a list of the DT/DBS studies discussed in this section. This is a representative rather than comprehensive list. Studies are organized into three broad categories based on methodology rather than the specific disease or DBS target.

**Table 2:** **List of articles on DT/DBS discussed in this review**.

Reference	Title	Patients	Disease
Anthofer et al. (2015)	DTI-based deterministic fiber tracking of the medial forebrain bundle	10	ET
Anthofer et al. (2014)	The variability of atlas-based targets in relation to surrounding major fiber tracts in thalamic deep brain stimulation	11	Depression
Barkhoudarian et al. (2010)	A role of diffusion tensor imaging in movement disorder surgery	3	PD, ET, Dystonia
Bhatia et al. (2012)	Diffusion tensor imaging to Aid subgenual cingulum target selection for deep brain stimulation in depression	59	Depression
Butson et al. (2006)	Predicting the effects of deep brain stimulation with diffusion tensor based electric field models	1	PD
Butson et al. (2007)	Patient-specific analysis of the volume of tissue activated during deep brain stimulation	1	PD
Calabrese et al. (2015a); Calabrese et al. (2015b)	Postmortem diffusion MRI of the human brainstem and thalamus for deep brain stimulator electrode localization	1	ET
Choi et al. (2015)	Mapping the “Depression switch” during intraoperative testing of subcallosal cingulate deep brain stimulation	9	Depression
Clelland et al. (2014)	Common cerebral networks associated with distinct deep brain stimulation targets for cluster headache	7	Cluster headace
Coenen et al. (2009)	Medial forebrain bundle stimulation as a pathophysiological mechanism for hypomania in subthalamic nucleus deep brain stimulation for Parkinson’s disease	6	PD
Coenen et al. (2011a)	A role of diffusion tensor imaging fiber tracking in deep brain stimulation surgery: DBS of the dentato-rubro-thalamic tract (drt) for the treatment of therapy-refractory tremor	1	Dystonia
Coenen et al. (2011b)	Individual fiber anatomy of the subthalamic region revealed with DTI—A concept to identify the DBS target for tremor suppression	1	PD
Coenen et al. (2012)	Diffusion tensor imaging and neuromodulation: DTI as key technology for deep brain stimulation	N/A	N/A
Coenen et al. (2014)	Modulation of the cerebello-thalamo-cortical network in thalamic deep brain stimulation for tremor	11	PD, ET, Dystonia
Copeland et al. (2014)	Deep brain stimulation of the internal globus pallidus for generalized dystonia associated with spinocerebellar ataxia type 1: a case report	1	Dystonia
Henderson (2012)	“Connectomic surgery”: diffusion tensor imaging (DTI) tractography as a targeting modality for surgical modulation of neural networks	N/A	N/A
Hunsche et al. (2013)	Tractography-guided stimulation of somatosensory fibers for thalamic pain relief	4	Chronic pain
Johansen-Berg et al. (2008)	Anatomical connectivity of the subgenual cingulate region targeted with deep brain stimulation for treatment-resistant depression	9	Depression
Klein et al. (2012)	The tremor network targeted by successful VIM deep brain stimulation in humans	12	PD
Kovanlikaya et al. (2014)	Treatment of chronic pain: diffusion tensor imaging identification of the ventroposterolateral nucleus confirmed with successful deep brain stimulation	1	Chronic pain
Lujan et al. (2011)	Axonal pathways linked to therapeutic and nontherapeutic outcomes during psychiatric deep brain stimulation	7	Depression
Lujan et al. (2013)	Tractography-activation models applied to subcallosal cingulate deep brain stimulation	1	Depression
McIntyre et al. (2004a); McIntyre et al. (2004b)	Uncovering the mechanism(s) of action of deep brain stimulation: activation, inhibition, or both	N/A	N/A
McNab et al. (2009)	Reduced limbic connections may contraindicate subgenual cingulate deep brain stimulation for intractable depression	1	Depression
Owen et al. (2007)	Preoperative DTI and probabilistic tractography in an amputee with deep brain stimulation for lower limb stump pain	1	Chronic pain
Owen et al. (2008)	Pre-operative DTI and probabilisitic tractography in four patients with deep brain stimulation for chronic pain	4	Chronic pain
Pouratian et al. (2011)	Multi-institutional evaluation of deep brain stimulation targeting using probabilistic connectivity-based thalamic segmentation	10	ET
Riva-Posse et al. (2014)	Defining critical white matter pathways mediating successful subcallosal cingulate deep brain stimulation for treatment-resistant depression	16	Depression
Rozanski et al. (2014)	Connectivity patterns of pallidal DBS electrodes in focal dystonia: a diffusion tensor tractography study	8	Dystonia
Said et al. (2014)	Correlation of diffusion tensor tractography and intraoperative macrostimulation during deep brain stimulation for Parkinson disease	17	PD
Schlaepfer et al. (2013)	Rapid effects of deep brain stimulation for treatment-resistant major depression	7	Depression
Schweder et al. (2010)	Chronic pedunculopontine nucleus stimulation restores functional connectivity	1	PD
Sedrak et al. (2008)	The role of modern imaging modalities on deep brain stimulation targeting for mental illness	15	PD, ET, Dystonia
Sudhyadhom et al. (2012)	Delineation of motor and somatosensory thalamic subregions utilizing probabilistic diffusion tractography and electrophysiology	5	ET
Sweet et al. (2014)	Fiber tractography of the axonal pathways linking the basal ganglia and cerebellum in Parkinson disease: implications for targeting in deep brain stimulation	14	PD
Torres et al. (2014)	Integrating diffusion tensor imaging-based tractography into deep brain stimulation surgery: a review of the literature	N/A	N/A

### Tract Stimulation Modeling

TSM (Figure 1B) refers to the practice of seeding DT from the region surrounding previously implanted DBS electrodes. The goal of TSM is to identify the population of brain connections that are likely to be modulated by a given DBS contact. This technique can be used to confirm that efficacious contacts are near or within a tract of interest. For example, Coenen et al. (2011b) performed TSM in a patient who received DBS for PD, and found that DT of the most efficacious contact yielded the expected DRT. The DRT was also identified by TSM of efficacious contacts in a patient with SCA1 dystonia by Copeland et al. (2014). Using similar methods, Kovanlikaya et al. (2014) found that TSM of efficacious contacts in a patient who received DBS for chronic pain demonstrated connections to the somatosensory cortex. TSM can also be used to identify pathways that lead to undesired side effects. Barkhoudarian et al. (2010) used TSM to demonstrate why stimulation of specific contacts at high voltages caused motor side effects in three patients who received DBS for movement disorders.

Another major use of TSM is to elucidate the activation patterns of successful vs. unsuccessful DBS for investigational applications. This exploratory (rather than confirmatory) use of TSM has been most widely used for depression, where the mechanism of DBS therapy is poorly understood. Johansen-Berg et al. (2008) generated a DT atlas of the subgenual cingulum from 17 healthy controls and then analyzed TSM of electrodes projected onto the atlas from nine patients who received DBS in the anterior cingulate cortex for refractory depression. They identified unique TSM patterns of efficacious contacts extending into frontal, limbic, and visceromotor regions. These results were built upon by Lujan et al. (2013) and Riva-Posse et al. (2014) in one and 17 patient(s) respectively. In both cases, TSM was compared between efficacious and non-efficacious contacts using simulated electric field models based on stimulation parameters. Both studies clearly showed different connectivity patterns of efficacious vs. non-efficacious contacts. Lujan et al. (2011) also performed a similar study in seven patients with refractory depression who received DBS of the ventral anterior internal capsule and ventral striatum. Once again, they found unique TSM patterns in responders and non-responders. Choi et al. (2015) performed intraoperative behavioral analysis on nine patients implanted for treatment-resistant depression, and found that contacts associated with acute positive mood changes were connected to the bilateral ventromedial frontal cortex and cingulate cortex. These TSM results lead the authors to suggest this specific connectivity pattern as a biomarker for effective DBS contact positioning.

Studies with similar methodologies have been conducted in patients who received DBS for cluster headache (Clelland et al., 2014), PD (Klein et al., 2012), primary dystonia (Rozanski et al., 2014), and chronic pain (Owen et al., 2007, 2008). In each case, authors analyzed TSM of efficacious vs. non-efficacious contacts in an effort to understand the neural networks that, when stimulated, lead to effective DBS therapy. These studies improve our understanding of the neurophysiologic underpinnings of brain diseases, and may eventually lead to more accurate and efficacious DBS targeting for the treatment of those diseases.

### Tract Proximity Analysis

TPA (Figure 1C) refers to retrospective analysis of the location of DBS contacts with respect to a specific tract of interest. This method can be used to answer the question of whether the proximity of a contact to a specific tract correlates with treatment efficacy, as well as for retrospective analysis of electrode targeting accuracy. TPA has been most commonly used in the study of DBS for movement disorders where putative tracts of interest (e.g., the DRT) are well described in the literature. Pouratian et al. (2011) performed TPA on a total of 10 DBS tremor patients and found that efficacious contacts were most likely to be associated with thalamic projections to the premotor cortex. Subsequent TPA studies have largely focused on the DRT as a putative target for the therapeutic effects of DBS in tremor patients. Sweet et al. (2014) found a non-significant trend in improved tremor control with increased contact proximity to the DRT in a study of 14 patients who received DBS for tremor-dominant PD. Similarly, Coenen et al. (2014) found a non-significant trend towards improved efficacy with increased proximity of the DRT to simulated contact electrical fields in 11 ET patients. Anthofer et al. (2014) also found that efficacious contacts were frequently near or within the DRT in a study of 10 ET patients, however no statistical analysis was performed. Finally, Calabrese et al. (2015b) were able to show a weak, but statistically significant correlation between treatment efficacy and contact proximity to the DRT, however, they used a high-resolution postmortem fiber atlas to generate the DRT model.

TPA has also been used to investigate DBS-related adverse effects in tremor patients including motor, sensory, and psychiatric side effects. Motor side effects have been studied both by Calabrese et al. (2015b), who showed no significant correlation between side effects and contact proximity to the DRT, and by Said et al. (2014) who show a non-significant trend towards greater contact voltage tolerability thresholds with increasing distance from the corticospinal tracts. Sensory side effects have also been studied, including work by Sajonz et al. (2015) who showed that contact proximity to the medial lemniscus was significantly correlated with hypogeusia and ageusia. Additionally, Coenen et al. (2009) suggested that stimulation of the median forebrain bundle was responsible for hypomania symptoms in a patient who received DBS for PD.

TPA studies serve as an important first step towards prospective targeting of DT-derived tracts with DBS electrodes. TPA has the benefit of allowing a specific tract hypothesis to be tested, however, unlike TSM, it requires *a priori* identification of a pathway of interest, which may not always be feasible. Once a tract of interest is identified for a given disease, TPA can be used to retrospectively test whether or not electrode proximity to that tract results in improved treatment efficacy. This in turn can provide initial evidence towards DT-based targeting of the tract of interest.

### Direct Tract Targeting

DTT (Figure 1D) refers to prospective targeting of DT-derived tracts with DBS electrodes. DTT requires not only *a priori* knowledge of a tract of interest, but also requires sufficient supporting evidence to justify targeting that tract in humans. Few putative tract targets meet these criteria, and as a result DTT studies are rare, and generally limited to cases where conventional methods are infeasible or ineffective. For example, Coenen et al. (2011a) describes successful DTT of the DRT in a patient with myoclonus dystonia. Conventional targeting, which employs intraoperative efficacy testing, was not possible because the patient’s head-dominant symptoms were obscured by the DBS stereotaxic head frame. Successful symptomatic control was achieved, and simulated electric fields from efficacious contacts were shown to overlap the DRT but not other adjacent tracts such as the corticospinal tracts. Schlaepfer et al. (2013) used DTT of the medial forebrain bundle to successfully treat six of seven patients with refractory depression, with the rationale that this tract cannot be reliably identified using conventional MRI images. Indeed, previous work by Bhatia et al. (2012) showed that DT based coordinates of the subgenual cingulum—a related DBS target for depression—differed significantly from coordinates derived from conventional T2-weighted MRI. Hunsche et al. (2013) used a hybrid DTT approach to target the posterior limb of the internal capsule in four patients with thalamic pain syndrome. The stereotaxic target was based on conventional targeting methods, but the electrode implantation trajectory was adjusted to provide at least 20 mm of overlap with DT of the spinothalamic tract. This approach lead to a 40% or greater pain relief in three of four patients.

In many ways, DTT is the ultimate goal of studies seeking to integrate DT into DBS planning. It is appealing because it allows targeting of structures that may not be visible using conventional methods, and it incorporates a mechanistic view of DBS as a neuromodulatory therapy. Unfortunately, to date there have been no controlled clinical trials comparing DTT to conventional targeting methods. Without such studies, it is impossible to say for certain whether DTT can improve DBS patient outcomes.

## Conclusions

While DT-based DBS targeting is still in its infancy, considerable progress has been made in incorporating DT into DBS surgery. These advances represent an exciting opportunity for both the DT and DBS communities. For DT researchers, DBS could become the second major clinical use for DT, the first being preoperative mapping of eloquent white matter pathways for brain tumor resection (Witwer et al., 2002; Nimsky et al., 2005). For the DBS community, DT provides a novel method for targeting structures that are not visible with conventional imaging methods, and may eventually help to elucidate the mechanisms underlying DBS therapy.

Interestingly, the three types of studies discussed here provide a reasonable workflow for DT-based investigation of novel DBS applications. For investigational DBS, TSM can be used to identify putative tracts of interest, which can in turn be validated using TPA, and then accurately targeted using DTT. This workflow is perhaps best exemplified by DBS of frontal lobe white matter pathways for refractory depression, where all three types of studies have been successfully performed (Coenen et al., 2012; Schlaepfer et al., 2013; Riva-Posse et al., 2014; Anthofer et al., 2015). If the issues facing DT-based DBS targeting are addressed, this workflow may become a valuable method for DT-based DBS target discovery, validation, and effective implementation.

Two major issues currently limit the potential of DT-based DBS targeting. First is the need for validation and standardization of DT, and second is the lack of randomized controlled trials. DT validation in humans is hindered by the inability to use “gold standard” methods like neuronal tracer studies. For this reason, animal studies, particularly those in non-human primates, are an important source of validation for DT (Schmahmann et al., 2007; Calabrese et al., 2014). However, no method is without limitations, and comparisons between DT and other neuronal mapping techniques are problematic because the data are fundamentally different (Thomas et al., 2014). Further, any information gleaned from animals will have to be extrapolated to humans, which reduces its utility for DBS planning. Interestingly, the integration of DT into DBS planning may itself provide important validation for DT. For example, comparisons of DT results with intraoperative microelectrode recording during DBS implantation has been used to validate DT-based segmentation of the thalamus (Sudhyadhom et al., 2012). The pathway towards standardization of DT is less clear, particularly since optimal methods are largely undefined. Knowledge of previous studies, like those discussed here, should help investigators make informed decisions on reasonable DT methods for their particular application.

Given these uncertainties it is no surprise that randomized controlled trials of DT-guided DBS have not been attempted to date. However, as with any emerging medical therapy, such trials will be essential for widespread adoption. The major challenge to the community will be in selecting the proper DBS application, DT methods, and the appropriate patient population for comparing DT-guided DBS to conventional methods.

## Author Contributions

EC performed the literature review and wrote the manuscript.

## Conflict of Interest Statement

The author declares that the research was conducted in the absence of any commercial or financial relationships that could be construed as a potential conflict of interest.
